# Potassium channel gene mutations rarely cause atrial fibrillation

**DOI:** 10.1186/1471-2350-7-70

**Published:** 2006-08-03

**Authors:** Patrick T Ellinor, Vadim I Petrov-Kondratov, Elena Zakharova, Edwin G Nam, Calum A MacRae

**Affiliations:** 1 Cardiovascular Research Center and Cardiac Arrhythmia Service Massachusetts General Hospital, Boston, Massachusetts

## Abstract

**Background:**

Mutations in several potassium channel subunits have been associated with rare forms of atrial fibrillation. In order to explore the role of potassium channels in inherited typical forms of the arrhythmia, we have screened a cohort of patients from a referral clinic for mutations in the channel subunit genes implicated in the arrhythmia. We sought to determine if mutations in KCNJ2 and KCNE1-5 are a common cause of atrial fibrillation.

**Methods:**

Serial patients with lone atrial fibrillation or atrial fibrillation with hypertension were enrolled between June 1, 2001 and January 6, 2005. Each patient underwent a standardized interview and physical examination. An electrocardiogram, echocardiogram and blood sample for genetic analysis were also obtained. Patients with a family history of AF were screened for mutations in KCNJ2 and KCNE1-5 using automated sequencing.

**Results:**

96 patients with familial atrial fibrillation were enrolled. Eighty-three patients had lone atrial fibrillation and 13 had atrial fibrillation and hypertension. Patients had a mean age of 56 years at enrollment and 46 years at onset of atrial fibrillation. Eighty-one percent of patients had paroxysmal atrial fibrillation at enrollment. Unlike patients with an activating mutation in KCNQ1, the patients had a normal QT_c _interval with a mean of 412 ± 42 ms. Echocardiography revealed a normal mean ejection fraction of 62.0 ± 7.2 % and mean left atrial dimension of 39.9 ± 7.0 mm. A number of common polymorphisms in KCNJ2 and KCNE1-5 were identified, but no mutations were detected.

**Conclusion:**

Mutations in KCNJ2 and KCNE1-5 rarely cause typical atrial fibrillation in a referral clinic population.

## Background

Atrial fibrillation (AF) is the most common clinical arrhythmia and accounts for approximately 3% of all medical admissions [1]. It is a major source of morbidity and mortality, due to chronic medication use, stroke and congestive heart failure[2,3]. While much is known of the mechanisms of AF initiation and maintenance[4], the molecular basis of variation in susceptibility to the arrhythmia is not known.

The identification of extended families with AF, and the mapping of discrete genetic loci in such families have raised the possibility that the biologic mechanisms underlying predisposition to the arrhythmia are accessible [5-7]. The first gene for AF was identified in a Chinese family with a persistent form of the arrhythmia. A mutation was found in the first transmembrane spanning domain (S140G) of the potassium channel gene, KCNQ1[8], previously implicated as a cause of Long QT syndrome, type 1 (LQT1). The majority of affected family members in the AF kindred exhibited no structural heart disease, but 3 patients were noted to have mild left ventricular dysfunction, and 9 of the 16 affected individuals had a prolonged QT interval. In cardiac myocytes, KCNQ1 associates with β subunits, such as KCNE1, to form the potassium channel carrying the I_Ks _current. Co-expression of the S140G mutant form of KCNQ1 with KCNE1 in COS-7 cells caused increased current density, as well as enhancement of channel activation and inactivation. These observations contrast with the consistent reduction in current density seen with those KCNQ1 mutations associated with LQT1, and also with the significant prolongation of the QT interval seen in many of the individuals in the AF family. This paradoxical result may reflect unique chamber-specific effects of the mutation or coupling of the channel with different partner proteins, but also serves to emphasize that our understanding of repolarization is incomplete. A second gain-of-function mutation in the KCNQ1 gene has since been found to cause AF in a child before birth, and in this case occurred along with the expected shortening of the QT interval[9]. Despite these two reports, mutations in KCNQ1 are an uncommon cause of AF in a typical population with the lone form of the arrhythmia[10].

Mutations in two other potassium channel subunit genes have been reported in association with AF. A single mutation in the KCNE2 gene, which encodes an β subunit common to both I_Ks _and I_Kr _complexes, was described in two patients with familial AF[11]. This sequence alteration partly segregated with disease in two kindreds and was not found in a large number of ethnically matched controls. *In vitro *data suggest that the sequence alteration (R27C) results in increased KCNQ1–KCNE2 inward and outward currents. Of note, this putative gain of function mutation would be predicted to shorten action potential duration, an electrophysiologic effect likely to favor the initiation and maintenance of AF. Another putative mutation in the KCNJ2 gene has been associated with isolated familial AF[12]. In this instance there is segregation of the mutation with the disease in a single kindred and there are *in vitro *data supporting a gain of function effect on the Kir 2.1 channel. While these data appear to strengthen the case for a common mechanism linking potassium channels with AF, the sequence alteration described results in a relatively conservative change at an amino acid level (V93I), and this very sequence is found in wild-type KCNJ2 protein in lower species suggesting that it may be a rare polymorphism. In addition, AF has been associated with rare gain of function mutations in KCNQ1, hERG and KCNJ2 causing the short QT syndrome and sudden death.

Interestingly, mice null for KCNE1 exhibit spontaneous AF implying at the very least a more complex mechanism than simple changes in ion conductance as the link between potassium channel genes and this arrhythmia. There are multiple KCNE isoforms, many, if not all, of which are expressed in the heart and thus are potential candidates for such a disease mechanism.

While a major genetic contribution to lone AF is clear, identifying the causal genes is likely to be an arduous task[10,13,14]. The paroxysmal and often asymptomatic nature of the phenotype usually precludes the identification of unaffected individuals and restricts many studies to affecteds only approaches, while overt genetic heterogeneity limits strategies based on aggregation of families. The small numbers of extended kindreds available restrict mapping studies and formal positional cloning efforts. In the face of these obstacles, direct sequencing in patient cohorts is the only feasible approach at present to confirm or refute potential candidate genes. We sought to determine whether mutations in KCNJ2 and KCNE1-5 play any role in more typical forms of this arrhythmia by screening a cohort of 96 patients with a family history of AF in at least one relative.

## Methods

### Clinical evaluation

All studies were performed with Institutional Review Board approval at Massachusetts General Hospital. Prior to any study procedures, written informed consent was obtained from the study patient. Serial patients with lone AF or AF/HTN referred to the arrhythmia service were enrolled between June 1, 2001 and January 6, 2005. Inclusion criteria were AF documented by electrocardiography, and an age less than or equal to 65 years. The exclusion criteria were structural heart disease as assessed by echocardiography, rheumatic heart disease, hyperthyroidism, myocardial infarction, or congestive heart failure.

Each patient underwent a physical examination and a standardized interview to identify past medical conditions, medications, symptoms and possible triggers for initiation of AF. The medical history of all first-degree relatives was also obtained. All patients were evaluated by 12-lead electrocardiogram (EKG), echocardiogram, and laboratory studies. Electrocardiograms and echocardiograms were interpreted using standard criteria.

From the cohorts with lone AF or AF/HTN, those patients reporting a family history of AF (a total of 96 individuals) underwent further genetic studies.

### Mutation detection

A blood sample (16 ml.) was obtained in acid citrate dextrose from every patient for the extraction of DNA. The isolation of DNA was performed directly from the original blood sample, or from a stable EBV-transformed lymphoblastoid cell line derived from this sample, using the Puregene DNA Purification Kit (Gentra Systems, Inc., Minneapolis, MN). Oligonucleotide primers for the coding regions of each gene were designed using the known cDNA and genomic sequence as follows:

KCNE1 (BC046224): CTTTAAGAGGTGTGCCTGGG, AATGTGATTAGAAAATCAGGTTGC,

KCNE2 (AF302095): CCGTTTTCCTAACCTTGTTCG, GACAATTTGGATTTGCCTCG,

KCNE3 (NM005472.3): ACAGCCAAACCATATCAGCC, ACGACCTCCCTTAACCGTG,

KCNE4(NM080671): GTTCCACAAACCTCGTGCTC, CCGCACTGTGAAAGTGAAAC,

KCNE5 (NM012282): GCTAGCTCGCTTCCCCTC, GAACCCTGGAAGGGAGTTG

KCNJ2(AF153820): Amplicon 1: CGAACATTCAAAACTGTTTCTCC, ACAGCAATTGGGCATTCATC, Amplicon 2: TCCGAGGTCAACAGCTTCAC

CCATGCCTTCCAGTATGACC, Amplicon 3: TCTGGTGTCCCCAATCAC,

GGGCCTCTGACCAACAGAC.

**Figure 1:**
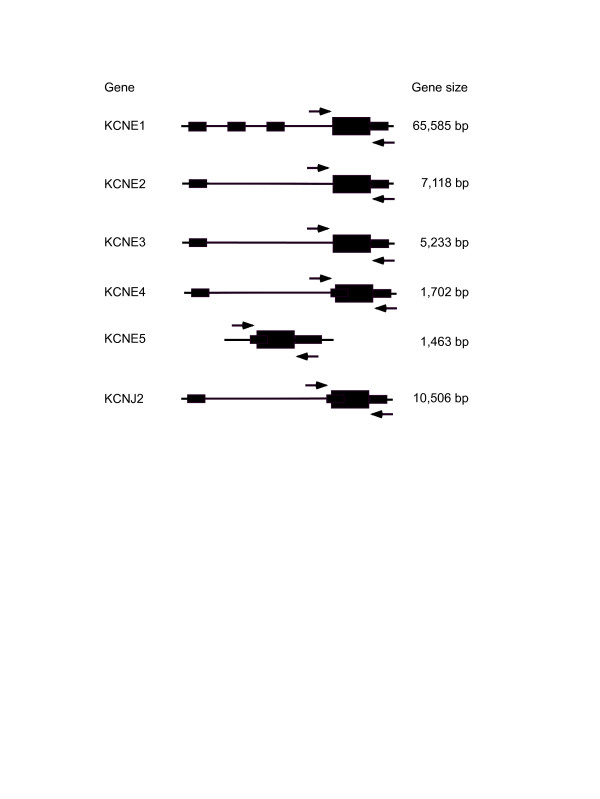
Size and Structure of the KCNE1-5 and KCNJ2 genes. Thin lines represent introns while exons are depicted as boxes. Small boxes are non-coding regions and large boxes are coding regions. Arrows indicate the location of PCR primers. The genomic size of each gene is presented to the right of each figure.

PCR was performed under the following conditions: 30–50 ng of genomic DNA was subjected to 35 cycles of amplification in a volume of 25 uL including 10 mM Tris HCl (pH 8.3), 50 mM KCl, 1.5 mM MgCl_2_, 200 mM dNTP, and 0.2 U Taq polymerase. For sequencing, PCR products were purified, and 25–50 ng of DNA was sequenced directly by use of the ABI PRISM dye terminator method (Model 377, Applied Biosystems, Foster City, California, USA). The gene structure, size and amplicons are illustrated in Figure 1.

### Statistical analyses

Continuous variables were tested for normality of distribution and two-sided t-tests were used for comparisons of means. The χ^2 ^test was used for categorical variables. P values of 0.05 or less were considered significant. All statistical analyses were performed using Intercooled STATA version 8.0.

## Results

During the study period a total of 252 patients with lone AF and 43 with AF/HTN were enrolled. From this total cohort, 83 patient with lone AF (33%) and 13 patients with AF/HTN (30%) reported a family history of AF in at least one first-degree relative. The clinical characteristics of these 96 patients are summarized in Table 1. The mean age at onset of AF was 46 years, and the age at enrollment was 56 years. Eighty-one percent of patients had paroxysmal AF at enrollment. The electrocardiograms of the study patients were notable for a normal mean PR of 176 ± 32.0 ms, QRS of 95.6 ± 15.3 ms, and QT_c _intervals of 412 ± 41.8 ms. Echocardiography of the study cohort revealed a mildly enlarged mean left atrial size of 39.9 ± 7.0 mm and a normal left ventricular ejection fraction, 62.0 ± 7.2 %.

**Table 1:** Baseline characteristics of the study cohort.

	**Mean ± SD or n (Percentage)**
Total	96
Age at enrollment (years)	56.1 ± 10.3
Age at onset of AF (years)	46.3 ± 12.2
Paroxysmal AF	78 (81.3)
Heart rate (bpm)	67 ± 14.1 bpm
Systolic blood pressure (mmHg)	126.3 ± 23.4
Diastolic blood pressure (mmHg)	77.3 ± 9.5
	
Electrocardiogram	
PR interval (ms)	176 ± 32.0
QRS interval (ms)	95.6 ± 15.3
QT_c _interval (ms)	412 ± 41.8
	
Echocardiogram	
Left atrial size (mm)	39.9 ± 7.0
Ejection fraction (percent)	62.0 ± 7.2
	
Ethnicity	
Caucasian	92 (96)
African-American	1 (1)
Asian	1 (1)
American Indian	1 (1)
Middle Eastern	1 (1)

The coding sequence of KCNJ2 and KCNE1-5 was screened for mutations by direct sequencing (Figure 1). While 17 polymorphisms were identified, no disease causing mutations were found (Table 2). Of the three polymorphisms that resulted in change in the protein sequence, all were found to be present in control populations.

**Table 2:** Observed polymorphisms in potassium channels.

**Gene**	**Exon**	**Polymorphism**	**Allele frequency**	**Reference**
KCNE1	1	G84A, S28S	0.0052	[17]
	1	G112A, G38S	0.24	[18]
	1	C158T, F53F	0.11	
	1	C240T, V80V	0.021	[19]
	1	G253A, **D85N**	0.021	[15], [20]
	1	G37A, Q13Q	0.0052	
KCNE3	1	T198C, F66F	0.09	[21]
	1	G248A, **R83H**	0.016	[16]
KCNE4	1	T81C, G27G	0.31	
	1	G69A, S23S	0.005	
	1	C264T, P88P	0.098	
	1	G435T, **E145D**	0.32	
	1	G471A, E157E	0.031	
	1	3'+18, G>C	0.073	
KCNE5	1	C97T, **P33S**	0.25	
	1	C207T, F69F	0.021	
KCNJ2	3	C1146T, L383L	0.13	

These are numbered based on the ATG using the cDNA sequence KCNE1 (BC046224), KCNE2 (AF302095), KCNE3 (NM005472.3), KCNE4 (NM080671), KCNE5 (NM012282), KCNJ2 (AF153820). Lower case letters refer to intronic base pairs and are located with respect to the corresponding intron-exon boundaries (5' or 3' +/- number of basepairs). Upper case letters refer to exonic polymorphisms. Polymorphisms that change the coding sequnce are indicated in bold. No polymorphisms were identified in KCNE2.

## Discussion

Potassium channel gene mutations resulting in increased repolarizing currents and shorter action potential duration are highly plausible as a cause of AF. Work in several large animal models has identified action potential shortening as one of the earliest effects of AF itself, and this 'electrical remodeling' is known to be a major factor in the maintenance of the arrhythmia and in its tendency to recur. In this context the identification of 'activating' mutations in the KCNQ1 gene in families with inherited forms of AF rapidly led to speculation that primary abnormalities of cardiac potassium currents might represent a common mechanism for the arrhythmia, and stimulated the search for mutations in other potassium channel subunit genes in affected individuals. Following up on previous isolated reports of mutations in the potassium channel subunit genes, KCNJ2 and KCNE1 we screened a cohort of 96 unrelated individuals with familial AF and found no evidence of causal KCNJ2 and KCNE1-5 mutations in 96 unrelated individuals. These data effectively exclude mutations in the KCNJ2 and KCNE1-5 gene as common causes of AF, and suggest that those families that do have mutations at this locus are unlikely to exhibit typical AF phenotypes.

Four polymorphisms that we identified resulted in an alteration of the coding sequence. The D85N variant of KCNE1 has previously been reported to decrease the I_Ks _current when co-expressed with KCNQ1 [15]. The R83H variant of KCNE3 was described in an individual with hypokalemic periodic paralysis; however, this variant was found in three of our patients none of whom had any prior episodes of paralysis and thus it appears likely that this is a rare polymorphism [16]. The functional effects of the E145D variant of KCNE4 and the P33S variant of KCNE5 are unknown.

While the genetic evidence supporting KCNQ1 mutations as a cause of AF is reasonably strong, in many of the other reported associations between AF and potassium channel gene mutations the absence of segregation data, the private nature of most of the sequence abnormalities described and inconsistent electrophysiologic findings all contribute to continued uncertainty. There are many potential reasons for these findings. Potassium channel gene mutations may simply represent a very rare cause of AF. However, it is also possible that the reported mutations are not acting through changes in ion channel conductance, but rather through other mechanisms, which may be shared across forms of AF. For example, the organization of the KCNQ1 genomic locus is only beginning to be understood, and effects on regional transcription or tissue-specific imprinting must be considered in addition to potential interactions within large multi-protein channel complexes. Finally, it is conceivable that the reported mutations in potassium channel genes are not causal in AF, but rather are genetically linked to the true causal genes. Ultimately, the identification of the causal genes in those rare families large enough for successful positional cloning efforts will offer insights into the true role of potassium channel genes in this disorder.

The current study has several intrinsic limitations. The cohort we studied was derived from referrals to an academic medical center and therefore may not be reflective of all cohorts with lone AF. Additionally, since our patients were recruited from a population of predominantly Northern European descent, mutations in potassium channel genes may still have a role in other ethnically groups.

## Conclusion

Mutations in potassium channels, KCNJ2 and KCNE1-5, rarely cause typical atrial fibrillation in a referral clinic population.

## Abbreviations

AF Atrial fibrillation

EKG Electrocardiogram

LQT1 Long QT syndrome, Type 1

## Competing interests

The author(s) declare that they have no competing interests.

## Authors' contributions

VIP, EAZ and EGN carried out the molecular genetic studies and performed the sequence analysis. PTE and CAM conceived of the study, and participated in its design and coordination, and wrote the manuscript. All authors read and approved the final manuscript.

## Pre-publication history

The pre-publication history for this paper can be accessed here:


